# Comparison of One-Person Technique and Two-Person Technique for Colonoscope Insertion: A Randomized Controlled Trial

**DOI:** 10.3390/jcm13113140

**Published:** 2024-05-27

**Authors:** Haegwang Shin, Jung Wan Choe, Seung Young Kim, Jong Jin Hyun, Sung Woo Jung, Young Kul Jung, Ja Seol Koo, Hyung Joon Yim

**Affiliations:** Department of Internal Medicine, Korea University Ansan Hospital, Ansan-si 15344, Republic of Korea

**Keywords:** colonoscopy, diagnostic techniques and procedures, quality, fatigue

## Abstract

**Background:** The one-person technique (OPT) for colonoscope insertion is recommended by professional societies and regarded as standard practice. However, the two-person technique (TPT) has shown several advantages over the OPT. The aim of this study was to evaluate the performance of the TPT compared to the OPT. **Methods:** In this prospective study, consecutive individuals presenting for outpatient colonoscopy were randomized to undergo colonoscopy by OPT or by TPT. The colonoscopies were performed by six endoscopists, two of whom were beginners, two with intermediate skills, and two who were experts. The primary endpoints were quality indicators for colonoscopy, including adenoma detection rate, cecal intubation rate, cecal insertion time, and total colonoscopy time. A secondary outcome was procedure tolerability, as assessed by both the patients and the endoscopists. **Results:** Two hundred and four subjects (117 males, mean age 54.3) were randomized to either one- (*n* = 102) or two-person (*n* = 102) colonoscopy. The adenoma detection rate was 30.4% in OPT group and 34.3% in TPT group. (*p* = 0.55). No significant differences between the two groups were found in terms of cecal intubation rate (98/102 vs. 98/102), insertion time (411 vs. 381 s), and total examination time (1426 vs. 1296 s). However, patients receiving the TPT had lower pain scores than patients receiving the OPT. Endoscopist fatigue measured with the FACIT-F was also significantly lower in the TPT group. **Conclusion:** The two-person colonoscopy method was not shown to be technically or clinically inferior. Rather, the TPT can improve patient tolerance and reduce endoscopist fatigue.

## 1. Introduction

Colonoscopy is considered the preferred modality for colorectal cancer screening because it has both diagnostic and therapeutic capabilities. The adenoma detection rate, cecum insertion time, and success rate of cecal insertion have become validated quality indicators for colonoscopy [1]. However, painful and unpleasant experiences are the most important factor in determining future compliance with colonoscopy from the patient’s point of view. Therefore, researchers continue to focus on methods that reduce patient discomfort and pain as well as improve polyp and adenoma detection and cecal insertion rates during colonoscopy [2,3].

The two methods for colonoscopy insertion are known as the one-person technique (OPT) and the two-person technique (TPT), independent of whether the insertion and withdrawal of the scope shaft are performed with or without the assistance of a nurse. Currently, the standard approach for colonoscopy insertion seems to favor the OPT, while the TPT is perceived as less commonly used [4]. There has been limited research addressing the TPT, and studies comparing it with the OPT are scarce [5,6]. Even among the studies that do exist, there have been no reports comparing the impact on patients’ pain levels between the OPT and TPT. Moreover, since endoscopist fatigue and musculoskeletal pain may influence the effectiveness of colonoscopy, further research is needed to determine which colonoscopy insertion method puts less physical burden on the endoscopist [7].

In this randomized controlled trial, we aimed to investigate which colonoscopy insertion method is better in terms of reducing patient discomfort and endoscopist fatigue as well as in terms of other parameters that represent high-quality colonoscopy.

## 2. Methods

### 2.1. Study Design

This randomized controlled prospective study was conducted at the Korea University Ansan Hospital, Korea. From December 2022 to March 2023, we recruited consecutive outpatients referred for screening or surveillance colonoscopy. This study was approved by the institutional review board of our hospital (2022AS0320, 26 November 2022). This trial has been registered with the clinical research information service, Republic of Korea (KCT 0008954). The exclusion criteria included sigmoidoscopy and therapeutic colonoscopy which were intended to resect neoplasia. Patients with a history of colorectal surgery, inflammatory bowel disease, polyposis syndromes, inaccessible stricture due to malignancy, and inadequate bowel preparation were also excluded.

### 2.2. Study Procedures

**Figure 1:**
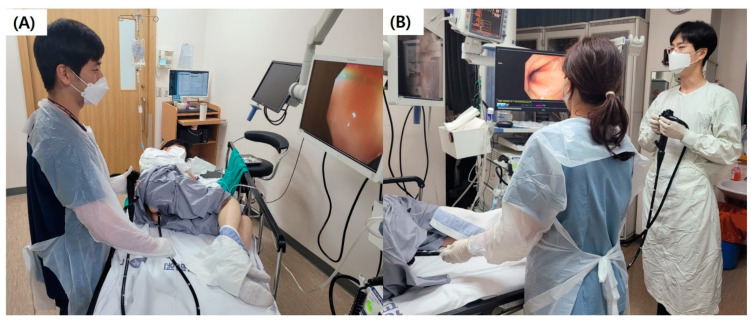
One-person technique and two-person technique. (**A**) One-person technique, in which the endoscopist independently maneuvers the endoscope during both insertion and retrieval. The endoscopist is responsible for adjusting the physical distance and movement direction in all dimensions. (**B**) Two-person technique, involving the collaboration of an assistant nurse. The assistant nurse holds the endoscope body at the insertion point and manages the forward and backward movement. Meanwhile, the endoscopist focuses on positioning the endoscope’s proximal tip at the colonic lumen’s center using control knobs.

Before undergoing a colonoscopy examination, patients were randomly assigned to one of two insertion methods, the OPT or the TPT. OPT stands for “one-person technique”, where the endoscopist operates the endoscope alone during insertion and retrieval, adjusting the physical distance for forward and backward movement as well as the direction of movement in all directions. TPT, on the other hand, is a “two-person technique”, where a nurse assistant holds the endoscope body at the insertion site and adjusts the physical distance for forward and backward movement, while the endoscopist positions the proximal tip of the endoscope at the center of the colonic lumen by manipulating the control knobs of the endoscope and instructs the nurse assistant to move the endoscope body forward or backward (Figure 1). Patients were unaware of which procedure they had been assigned to, while the endoscopist and assistant were informed of the assigned procedure before the colonoscopy. The colonoscopy examinations were performed by a total of six endoscopists. They were classified into beginner, intermediate, and expert groups, with two endoscopists in each group. Based on the consideration of learning curves for colonoscopy examinations performed using the OPT, two endoscopists (L.J.S., J.C.M.) with 50 or fewer OPT colonoscopy experiences were classified as beginners, while two endoscopists (C.J.W., K.D.W.) with over 200 colonoscopy experiences were defined as experts in endoscopy. Additionally, the final two endoscopists (K.S.S., H.S.H.) with colonoscopy experience between 50 and 200 procedures were classified as intermediate specialists [8]. All endoscopists had received training in the OPT, with little to no experience in the TPT, either completely absent or fewer than 10 cases. Three assistant nurses (J.S.Y., H.Y.S., and J.H.W.) participated in the procedures, but they also had little to no prior experience with the TPT. They simply followed the endoscopist’s instructions to move the endoscope forward or backward.

### 2.3. Sedation for Colonoscopy

Patients were sedated intravenously using midazolam and propofol. The doses were administered uniformly according to the guidelines, starting with midazolam 0.03 mg/kg and propofol 0.5 mg/kg one minute before the start of the exam, and the exam was performed under adequate sedation. We continuously monitored the oxygen saturation and heart rate of the patients during the examination. In cases where the patients complained of pain, additional doses of midazolam at 0.01 mg/kg and/or propofol at 20 mg were administered at the discretion of the endoscopist, with no limit on the number of administrations, while closely monitoring cardiorespiratory function.

### 2.4. Primary and Secondary Endpoints

Primary endpoints were colonoscopy performance indicators, including polyp and adenoma detection rates, cecal insertion rate, insertion time, and total procedure time were investigated. A colon polyp was defined as a small growth of tissue formed on the inner lining of the colon. If a colon polyp exhibited dysplasia, it was categorized as an adenoma. Cecum insertion failure was defined as the inability to reach the cecum within 20 min using the initially assigned insertion method. Another endoscopist, who was not involved in this study, conducted the failed colonoscopy with their preferred insertion method. If, despite these attempts, cecum insertion was still not achieved within an additional 20 min, it was classified as a final insertion failure. Instead, stool examination and CT were used as alternatives. Insertion time referred to the duration for the colonoscope to reach the cecum, and total procedure time was the overall duration from the insertion of the colonoscope to its removal after completing the examination.

Secondary endpoints included the total dosage of the sedative agent and opioid as well as additional dose administration frequency. Through this, we indirectly assessed the level of pain experienced by patients. The occurrence of adverse events such as hypoxia and hypotension during the examination was also monitored. Pain scores reported by patients and the endoscopist’s perceived fatigue were surveyed through questionnaires after the procedure. Pain scores for patients undergoing colonoscopy were assessed after complete recovery from sedation using a numerical rating scale that ranged from 0 (indicating no pain) to 5 (indicating the highest level of pain). Additionally, the evaluation of the endoscopist’s physical fatigue was conducted using the Functional Assessment of Chronic Illness Therapy Fatigue (FACIT-F) questionnaire, with scores ranging from 0 to 52 [8]. Additionally, to investigate differences in the two insertion methods based on the endoscopist’s experience, subgroup analysis was conducted.

### 2.5. Sample Size Calculation and Statistical Analysis

The sample size for the current non-inferiority trial was determined based on a baseline adenoma detection rate of 30% in the OPT, which was derived from a historical internal quality assurance program [9]. Taking into account an alpha level of 0.05, with beta 0.2, delta 0.05, and a 10% dropout rate, 112 subjects had to be included in each arm. Categorical variables are presented as frequencies and percentages. Continuous variables are presented as the mean (±SD) or median with range. For the comparison of the OPT and the TPT, chi-square tests or Fisher’s exact tests were performed for categorical variables, and two-tailed *t*-tests were performed for continuous variables. Statistical significance was set at *p* < 0.05. All statistical analyses were performed using SPSS version 21.0 for Windows (SPSS Inc., Chicago, IL, USA).

## 3. Results

### 3.1. Baseline Characteristics

**Figure 2:**
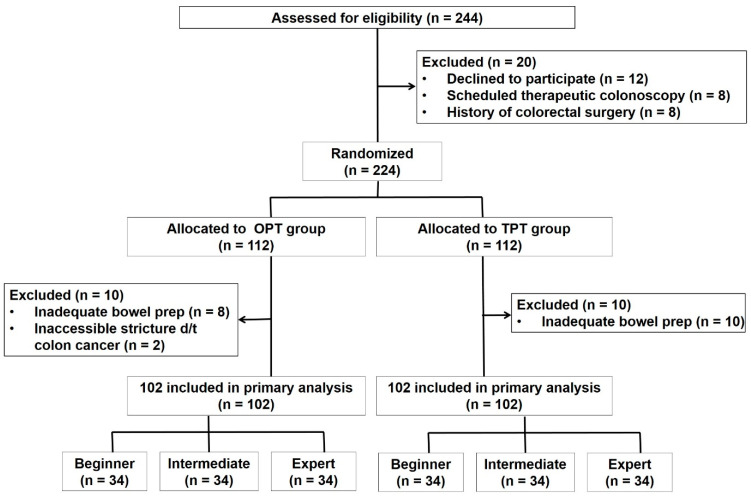
Flowchart of study participants. OPT, one-person technique; TPT, two-person technique; prep, preparation.

A schematic of the study design is depicted in Figure 2. Two hundred and forty-four eligible patients were initially enrolled, but after excluding 20 individuals based on exclusion criteria, 224 patients underwent simple randomization using a computer-generated sequence. They were classified into the OPT group and the TPT group in a 1:1 ratio. Patients with difficulties in endoscopic entry due to inadequate bowel preparation or challenges arising from colorectal cancer-related strictures were excluded from this study. As a result, there were 102 patients in the OPT group and 102 patients in the TPT group. There were no significant differences observed between the two groups in terms of age, body mass index, and gender (Table 1). There were also no significant differences in terms of underlying conditions such as cardiovascular disease, diabetes mellitus, chronic kidney disease, liver disease, malignancy, pulmonary disease, and neurologic disease. In both groups, 62% of the patients had a history of previous colonoscopy. Additionally, all patients reported successful cecal insertion during previous examinations, with full inspection of the colon being carried out. In terms of past surgical history, particularly surgeries that could influence colonoscopy insertion, such as upper gastrointestinal, hepatobiliary, and gynecological surgeries, there was a slight tendency for more occurrences in the TPT group, which was not statistically significant.

### 3.2. Primary Outcomes

Both the OPT and the TPT achieved cecal insertion rates of 96%, with four failures in each group (Table 2). Among the four patients in whom cecal insertion could not be achieved with the OPT, cecal insertion finally succeeded in three cases, resulting in a 99% (101/102) final cecal insertion rate. Similarly, among the four patients in whom cecal insertion initially failed with the TPT, successful cecal insertion was achieved in two cases, resulting in a final cecal insertion rate of 98% (100/102). Cecal insertion time and total procedure time trended slightly shorter in the TPT group compared to the OPT, but with no significant difference. In the TPT group, the polyp detection rate was higher compared to the OPT (40.2% vs. 44.1%, *p* = 0.56). Similarly, the adenoma detection rate in the TPT group was also higher (30.4% vs. 34.3%, *p* = 0.55), but these differences did not reach statistical significance.

### 3.3. Secondary Outcomes

The usage of sedative agents such as propofol and midazolam, as well as the amount of pethidine used for pain control, was lower in the TPT group compared to the OPT group. Additional administration frequency of sedation agents was less frequent in the TPT group, but these differences were not significant. Complications during endoscopic procedures, including a decrease in SaO_2_ and paradoxical responses to sedative agents, occurred in 10 cases in the OPT group and 7 cases in the TPT group. The patient pain score for colonoscopy was 2.39 in the OPT group and 2.15 in the TPT group, indicating that patients in the TPT group reported significantly less pain. Additionally, the FACIT-F score also showed that the TPT had notably lower scores than the OPT, suggesting lower operator fatigue associated with the TPT.

### 3.4. Subgroup Analysis according to Endoscopist Experience Levels

A total of eight cases of cecal insertion failure occurred in the beginner and intermediate groups. In contrast, there were no instances of cecal insertion failure among experts (Table 3). In both the OPT and TPT groups, higher levels of endoscopic experience were associated with shorter insertion times and total procedure times. Furthermore, there was a trend in both groups in which higher levels of endoscopist experience correlated with lower total doses of midazolam and propofol used as well as fewer instances of additional administration. As well, it was observed that both patient pain and endoscopist discomfort decreased with higher levels of endoscopist experience. In the OPT group, there were significant differences in insertion time, total procedure time, adenoma detection rate, patient pain score, and endoscopist fatigue based on the endoscopist’s experience. However, in the TPT group, no significant differences were observed. Specifically, in terms of adenoma detection, higher experience levels led to greater detection rates in the OPT group. In contrast, within the TPT group, there were no significant differences in polyp detection rates among the three experience levels. Interestingly, the beginner and intermediate groups showed higher polyp detection rates compared to the expert group.

## 4. Discussion

In this study, clinical outcomes were compared based on two different colonoscopy insertion methods. Our results showed that the TPT demonstrated a tendency towards shorter cecal insertion time, higher polyp and adenoma detection rates, and less use of sedative agents compared to the OPT. Moreover, the TPT significantly reduced the levels of patient pain and the fatigue burden on the endoscopist.

During the initial stages of the development of colonoscopy as a procedure, nurse assistants aided in the insertion process due to the lengthy and rigid nature of endoscopes, along with their challenging maneuverability [6]. However, as colonoscopy equipment has become lighter and more flexible and with advances in endoscopic techniques among endoscopists, the OPT method has emerged as the predominant approach for performing colonoscopy. Conversely, the TPT method is rarely practiced in clinical settings. For these reasons and for its clinical effectiveness, the OPT is endorsed by professional societies and is considered the established standard of practice in the United States [4]. Research on the OPT and TPT techniques for colonoscopy examinations is quite limited. According to a retrospective study published in Norway in 2011, only 17% of colonoscopy practitioners in Norway employed the TPT, while 83% conducted colonoscopy examinations using the OPT approach [6]. In terms of cecal insertion rates, no significant differences were observed between the TPT and OPT. However, TPT exhibited advantages such as shorter procedure times and reduced use of sedatives and analgesics. These findings are consistent with the results presented here. However, that study was a retrospective investigation conducted with non-randomized selection of the TPT and OPT procedural methods. Furthermore, as surveys regarding pain during colonoscopy were conducted after patients had returned home, there is a limitation associated with potential recall bias. In another study, the benefits of the OPT were emphasized, which stands in contrast to our findings. These included a shorter insertion time (6.8 vs. 8.0 min) along with decreased patient discomfort and a reduced scope length to the cecum [10]. The authors suggested that the OPT could provide a better sense of colon resistance and redundancy, potentially minimizing forceful insertion, reducing patient discomfort, and lowering complication rates. However, this study also had limitations as it was conducted by a single experienced endoscopist and an assistant, with a small sample size, making it challenging to generalize the results. Additionally, all procedures were performed without sedation, which differs from the current practice in many countries.

The strength of this study lies in its undertaking of a prospective randomized comparative design involving 204 patients for the two procedure methods. Moreover, we evaluated not only the verified quality indicators for colonoscopy but also assessed various aspects of the techniques through surveys administered to both the patients and the endoscopists. This study revealed several advantages of the TPT compared to the OPT. Firstly, the TPT was observed to be less influenced by endoscopist proficiency, particularly in terms of factors such as cecal insertion time, total procedure time, and adenoma detection rate. Achieving competence in performing colonoscopy with the OPT has been reported to require a minimum of 100–200 procedures and a significant time investment [11]. Although there is a lack of existing research on the learning curve for the TPT, this study demonstrates that even among endoscopists who are relatively inexperienced with the TPT, the TPT yields comparable cecal insertion rates and shorter cecum insertion times compared to the OPT. Additionally, TPT training is relatively straightforward for both endoscopist trainees and assistant nurses. For endoscopy trainees, TPT training focuses on positioning the proximal tip of the endoscope at the center of the colonic lumen by manipulating the up/down and left/right control knobs. In the OPT, where the left/right control knob is typically not used, torque is applied to turn the colonoscope left or right. In the TPT, using the left/right control knobs instead of torque is the primary difference, but it is not particularly challenging to manipulate the up/down and left/right control knobs simultaneously. Additionally, for assistant nurses, advanced training may not be necessary since basic actions like pushing forward and pulling back the endoscope body are directed by the physician’s instructions. However, it is crucial for them to avoid pushing forward independently, especially when faced with resistance or unclear visibility of the lumen during endoscope insertion, as this could lead to perforation. Secondly, the TPT also demonstrated advantageous aspects in terms of endoscopist fatigue. In both the OPT and TPT groups, as the operator’s proficiency improved, there was a reduction in endoscopist fatigue. Notably, in all groups, namely among beginners, intermediate specialists, and experts, the TPT caused significantly less fatigue compared to the OPT. The act of pushing, pulling, and exerting torque on the scope with a single hand in the OPT can impose stress on the wrist, elbow, and shoulder joints [12]. This, in turn, diminishes procedural efficiency and can impede precise examinations. In contrast, the TPT involves a relatively two-handed manipulation of the endoscope control unit, with adjustments to the knobs executed gently using the fingers. As a consequence, endoscopist fatigue could be reduced, allowing for the potential of conducting examinations with heightened accuracy. Thirdly, the TPT was associated with quicker recovery times and lower sedative doses. This not only reduces the financial burden from decreased sedative agent usage but also has the potential to expedite turnover in the recovery room. Consequently, it could help alleviate some of the cost-related disadvantages associated with space and the requirement for assistants in TPT procedures [13]. Lastly, in the TPT, where the endoscopist and assistant jointly view the screen during the insertion and retrieval of the endoscope, it was anticipated that the polyp detection rate would be higher compared to the OPT, where the procedure is carried out alone. Although no statistical significance was observed, it was confirmed that the polyp and adenoma detection rates were higher in the TPT group in this study. Additionally, during the procedure, the continuous support of the assistant in holding the endoscope reduced the risk of missing polyps during polypectomy while also likely contributing to a favorable effect on the total procedure time.

Nevertheless, there exist potential drawbacks to the TPT as well. Firstly, the implementation of the TPT necessitates additional personnel and allocated space [14]. Secondly, the endoscopist must continuously perceive the degree of resistance during endoscope insertion and be capable of adjusting resistance adequately by straightening loops in response to resistance, aiming to minimize patient discomfort and potential complications resulting from the procedure [15]. However, in the TPT, the endoscopist is unable to directly sense the resistance during endoscope insertion and instead relies on assistant cues for guidance. Lastly, while for the OPT, coordinated actions such as endoscope advancement, knob-controlled angulation, and endoscope torque enable relatively smooth navigation through challenging flexures, in the TPT, the endoscopist and assistant need to perform synchronized actions with the same intent to achieve comparable results. When the endoscopist and assistant have differing interpretations, the procedure becomes significantly more challenging.

This study has some limitations. First, it was not possible to achieve a double-blind design. While efforts were made to conceal the insertion method from the patients, it was not possible to do so for the endoscopists. Consequently, this study did not adhere to a double-blind approach, potentially introducing bias into the results. Second, the assessment of endoscopist fatigue involved administering the FACIT-F questionnaire after each procedure. However, due to variations in the rate of fatigue changes among endoscopists and the subjectivity of the evaluation method, accurately reflecting the fatigue levels associated with each procedure could be considered limited. Thirdly, this study was conducted with a relatively small number of patients within a single center, with procedures carried out exclusively by only six endoscopists at that facility. Consequently, the applicability of the results to other medical institutions or endoscopists may be limited. Therefore, subsequent multicenter studies involving a larger patient cohort are necessary to compare outcomes based on the techniques of various endoscopists. Finally, the influence of assistants was not accounted for in this study. Although three nurses participated, their roles were not stratified based on experience. Assistant nurses primarily managed the advancement and withdrawal of the scope, and their skills might not have significantly influenced the research outcomes. However, they could still have impacted the procedures by providing information to the endoscopist regarding the presence of loops or resistance during insertion. Therefore, further research categorized according to the experience of assistant nurses could be conducted based on a larger number of cases.

In conclusion, the TPT has demonstrated non-inferiority to the OPT by exhibiting shorter insertion times, shorter procedure durations, reduced sedation requirements, and higher polyp and adenoma detection rates. Furthermore, the TPT significantly alleviated patient discomfort and reduced musculoskeletal fatigue among endoscopists. Therefore, considering its procedural and clinical benefits, the TPT should be regarded not as a technique to be avoided, but rather as an approach to be embraced and skillfully adopted by endoscopic specialists.

## Figures and Tables

**Table 1:** Baseline characteristics of enrolled patients.

	OPT (*N* = 102)	TPT (*N* = 102)	*p*-Value
Age	54.0 ± 14.1	54.7 ± 13.6	0.88
Sex (male/female)	60 (58.8%)/42 (41.2%)	57 (55.9%)/45 (44.1%)	0.67
Height	164.0 ± 8.8	163.0 ± 9.0	0.34
Body weight	64.1 ± 11.3	65.0 ± 13.6	0.98
BMI	23.8 ± 3.2	24.3 ± 3.5	0.25
Prior abdominal/pelvic surgery			0.34
None	74 (72.5%)	69 (67.6%)	
Stomach	5 (4.9%)	6 (5.9%)	
Colon	11 (10.8%)	10 (9.8%)	
Hepatobiliary	2 (2.0%)	5 (4.9%)	
OBGY	6 (5.9%)	12 (11.8%)	
Underlying disease			
Cardiovascular	32 (31.4%)	33 (32.3%)	0.88
DM	25 (24.5%)	20 (19.6%)	0.40
CKD	3 (2.9%)	5 (4.9%)	0.72
Liver disease	3 (2.9%)	2 (2.0%)	1
Malignancy	2 (2.0%)	6 (5.9%)	0.28
Pulmonary disease	6 (5.9%)	4 (3.9%)	0.52
Neurologic disease	6 (5.9%)	1 (1.0%)	0.12

Values are n (%) or mean ± SD. OPT, one-person technique; TPT, two-person technique; BMI, body mass index; OBGY, obstetrics and gynecology; DM, diabetes mellitus; CKD, chronic kidney disease.

**Table 2:** Clinical outcomes in OPT group and TPT group.

	OPT (*N* = 102)	TPT (*N* = 102)	*p*-Value
Cecal insertion success with initial technique	98/ 102 (96.1%)	98/102 (96.1%)	1.00
Final cecal insertion success	101/102 (99.0%)	100/102 (98.0%)	1.00
Cecal insertion time (s)	410.7 ± 212.2	381.4 ± 217.4	0.18
Total procedure time	1425.8 ± 723.6	1296.3 ± 624.9	0.24
Polyp detection rate	41/102 (40.2%)	45/102 (44.1%)	0.56
Adenoma detection rate	31/102 (30.4%)	35/102 (34.3%)	0.55
Mean medication dose			
Propofol	89.2 ± 36.6	79.3 ± 46.5	0.01
Midazolam	3.56 ± 1.21	3.23 ± 1.33	0.09
Pethidine	28.0 ± 6.02	25.2 ± 10.4	0.18
Additional sedative administration frequency	2.3 ± 1.6	1.9 ± 1.6	0.06
Complications	10/102 (9.8%)	7/ 102 (6.9%)	0.09
Recovery time	44.4 ± 19.1	37.1 ± 18.9	<0.001
Patient pain score	2.39 ± 1.79	2.15 ± 1.53	0.04
FACIT-F by endoscopist	36.8 ± 7.2	22.8 ± 9.8	<0.001

Values are n (%) or mean ± SD. OPT, one-person technique; TPT, two-person technique; FACIT-F, Functional Assessment of Chronic Illness Therapy Fatigue.

**Table 3:** Subgroup analysis of the one-person technique group and two-person technique group according to endoscopist experience levels.

**One-Person Technique**									
	**Beginner** **(*N* = 34)**			**Intermediate** **(*N* = 34)**			**Expert** **(*N* = 34)**		***p*-Value**
Initial cecal intubation rate	91.2% (31/34)			97.1% (33/34)			100% (34/34)		1.00
Cecal insertion time (s)	479.5 ± 222.2			410.7 ± 167.3			342.4 ± 213.5		0.03
Total procedure time (s)	1656.6 ± 719.1			1421.9 ± 892.4			1201.6 ± 478.7		0.04
Adenoma detection rate	20.6% (7/34)			29.4% (10/34)			41.2% (14/34)		0.03
Propofol	95.2 ± 41.6			89.3 ± 32.7			83 ± 25.6		0.39
Midazolam	3.77 ± 1.24			3.58 ± 1.04			3.38 ± 1.29		0.37
Pethidine	30.6 ± 8.0			27.5 ± 6.5			26.1 ± 5.6		0.54
Additional sedative administration frequency	2.6 ± 1.9			2.3 ± 1.2			1.9 ± 1.1		0.19
Complications	6 (17.6%)			3 (8.8%)			1 (3%)		0.45
Recovery time	48.1 ± 21.2			45.0 ± 9.2			40.2 ± 20.0		0.39
Patient pain score	2.72 ± 2.03			2.34 ± 1.57			2.11 ± 1.34		0.04
FACIT-F by endoscopist	45.6 ± 12.8			36.9 ± 9.2			27.9 ± 8.1		<0.001
**Two-person technique**									
	**Beginner** **(*N* = 34)**			**Intermediate** **(*N* = 34)**			**Expert** **(*N* = 34)**		***p*-value**
Initial cecal intubation rate	94.1% (32/34)			94.1% (32/34)			100% (34/34)		0.50
Cecal insertion time (s)	414.8 ± 252.4			386.7 ± 161.3			345.2 ± 86.0		0.06
Total procedure time (s)	1393.0 ± 513.9			1286.2 ± 858.8			1210.3 ± 276.1		0.10
Adenoma detection rate	38.2% (13/34)			35.2% (12/34)			29.4% (10/34)		0.58
Propofol	81.1 ± 48.5			78.5 ± 47.5			77.9 ± 28.0		0.25
Midazolam	3.37 ± 1.48			3.20 ± 0.82			3.12 ± 1.27		0.37
Pethidine	27.6 ± 3.6			24.5 ± 5.7			23.5 ± 4.3		0.58
Additional sedative administration frequency	2.3 ± 1.4			1.80 ± 1.2			1.6 ± 0.8		0.43
Complications	4 (11.8%)			1 (2.9%)			2 (5.8%)		0.11
Recovery time	40.1 ± 18.5			37.0 ± 22.2			34.3 ± 11.4		0.07
Patient pain score	2.35 ± 1.60			2.19 ± 1.47			1.91 ± 1.47		0.08
FACIT-F by endoscopist	25.2 ± 10.3			23.2 ± 9.3			20.0 ± 7.4		0.58
	**Beginner**			**Intermediate**			**Expert**		
	**OPT** **(*N* = 34)**	**TPT** **(*N* = 34)**	***p*** **-value**	**OPT** **(*N* = 34)**	**TPT** **(*N* = 34)**	***p*** **-value**	**OPT** **(*N* = 34)**	**TPT** **(*N* = 34)**	***p*** **-value**
Initial cecal intubation rate	91.2% (31/34)	94.1% (32/34)	0.64	97.1% (33/34)	94.1% (32/34)	0.55	100% (34/34)	100% (34/34)	1.00
Cecal insertion time (s)	479.5 ± 222.2	414.8 ± 252.4	0.27	410.7 ± 167.3	386.7 ± 161.3	0.54	342.4 ± 213.5	345.2 ± 86.0	0.94
Total procedure time (s)	1656.6 ± 719.1	1393.0 ± 513.9	0.09	1421.9 ± 892.4	1286.2 ± 858.8	0.53	1201.6 ± 478.7	1210.3 ± 276.1	0.92
Adenoma detection rate	20.6% (7/34)	38.2% (13/34)	0.11	29.4% (10/34)	35.2% (12/34)	0.27	41.2% (14/34)	29.4% (10/34)	0.31
Propofol	95.2 ± 41.6	81.1 ± 48.5	0.02	89.3 ± 32.7	78.5 ± 47.5	0.02	83 ± 25.6	77.9 ± 28.0	0.04
Midazolam	3.77 ± 1.24	3.37 ± 1.48	0.04	3.58 ± 1.04	3.20 ± 0.82	0.10	3.38 ± 1.29	3.12 ± 1.27	0.40
Pethidine	30.6 ± 8.0	27.6 ± 3.6	0.06	27.5 ± 6.5	24.5 ± 5.7	0.10	26.1 ± 5.6	23.5 ± 4.3	0.22
Additional sedative administration frequency	2.6 ± 1.9	2.3 ± 1.4	0.46	2.3 ± 1.2	1.80 ± 1.2	0.09	1.9 ± 1.1	1.6 ± 0.8	0.20
Complications	6 (17.6%)	4 (11.8%)	0.49	3 (8.8%)	1 (2.9%)	0.30	1 (3%)	2 (5.8%)	0.35
Recovery time	48.1 ± 21.2	40.1 ± 18.5	0.01	45.0 ± 9.2	37.0 ± 22.2	0.02	40.2 ± 20.0	34.3 ± 11.4	0.04
Patient pain score	2.72 ± 2.03	2.35 ± 1.60	0.02	2.34 ± 1.57	2.19 ± 1.47	0.04	2.11 ± 1.34	1.91 ± 1.47	0.55
FACIT-F by endoscopist	45.6 ± 12.8	25.2 ± 10.3	<0.001	36.9 ± 9.2	23.2 ± 9.3	<0.001	27.9 ± 8.1	20.0 ± 7.4	<0.001

Values are n (%) or mean ± SD. OPT, one-person technique; TPT, two-person technique; FACIT-F, Functional Assessment of Chronic Illness Therapy Fatigue.

## Data Availability

Dataset available on request from the authors.
